# Research progress on the prevention and treatment of hyperuricemia by medicinal and edible plants and its bioactive components

**DOI:** 10.3389/fnut.2023.1186161

**Published:** 2023-06-12

**Authors:** Wang Cheng-yuan, Dai Jian-gang

**Affiliations:** Department of food science, School of Environment and Resources, Chongqing Technology and Business University, Chongqing, China

**Keywords:** hyperuricemia, medicinal and edible plants, xanthine oxidase, uric acid transporters, bioactive components, mechanisms of action

## Abstract

Hyperuricemia is another common metabolic disease, which is considered to be closely related to the development of many chronic diseases, in addition to the “three highs.” Currently, although drugs show positive therapeutic effects, they have been shown to produce side effects that can damage the body. There is growing evidence that medicinal and edible plants and their bioactive components have a significant effect on hyperuricemia. In this paper, we review common medicinal and edible plants with uric acid-lowering effects and summarize the uric acid-lowering mechanisms of different bioactive components. Specifically, the bioactive components are divided into five categories: flavonoids, phenolic acids, alkaloids, polysaccharides, and saponins. These active substances exhibit positive uric acid-lowering effects by inhibiting uric acid production, promoting uric acid excretion, and improving inflammation. Overall, this review examines the potential role of medicinal and edible plants and their bioactive components as a means of combating hyperuricemia, with the hope of providing some reference value for the treatment of hyperuricemia.

## 1. Introduction

Hyperuricemia (HUA) is a metabolic disease caused by abnormal purine metabolism in the body. Clinically, HUA is a higher-than-normal saturated concentration of urate in the blood in the fasting state. The standard for men is >7 mg/dL, for women >6 mg/dL, and for adolescents and children >5.5 mg/dL (1). Uric acid (UA) is synthesized by the body’s organs through the conversion of purines produced by their own nucleic acid metabolism and/or food purines (2). Excess uric acid can be deposited in the kidneys and synovial fluid to form monosodium urate crystals, causing inflammatory reactions and gout (3). With the change in people’s diet, the incidence of Hpegloticasepeople’s dietUA is increasing year by year in the world, especially in China and the United States (4, 5). Studies have found that high uric acid not only causes gout, but also causes other metabolic diseases, including cardiovascular disease, hypertension, and intestinal flora disorders (1, 6, 7).

**Figure 1:**
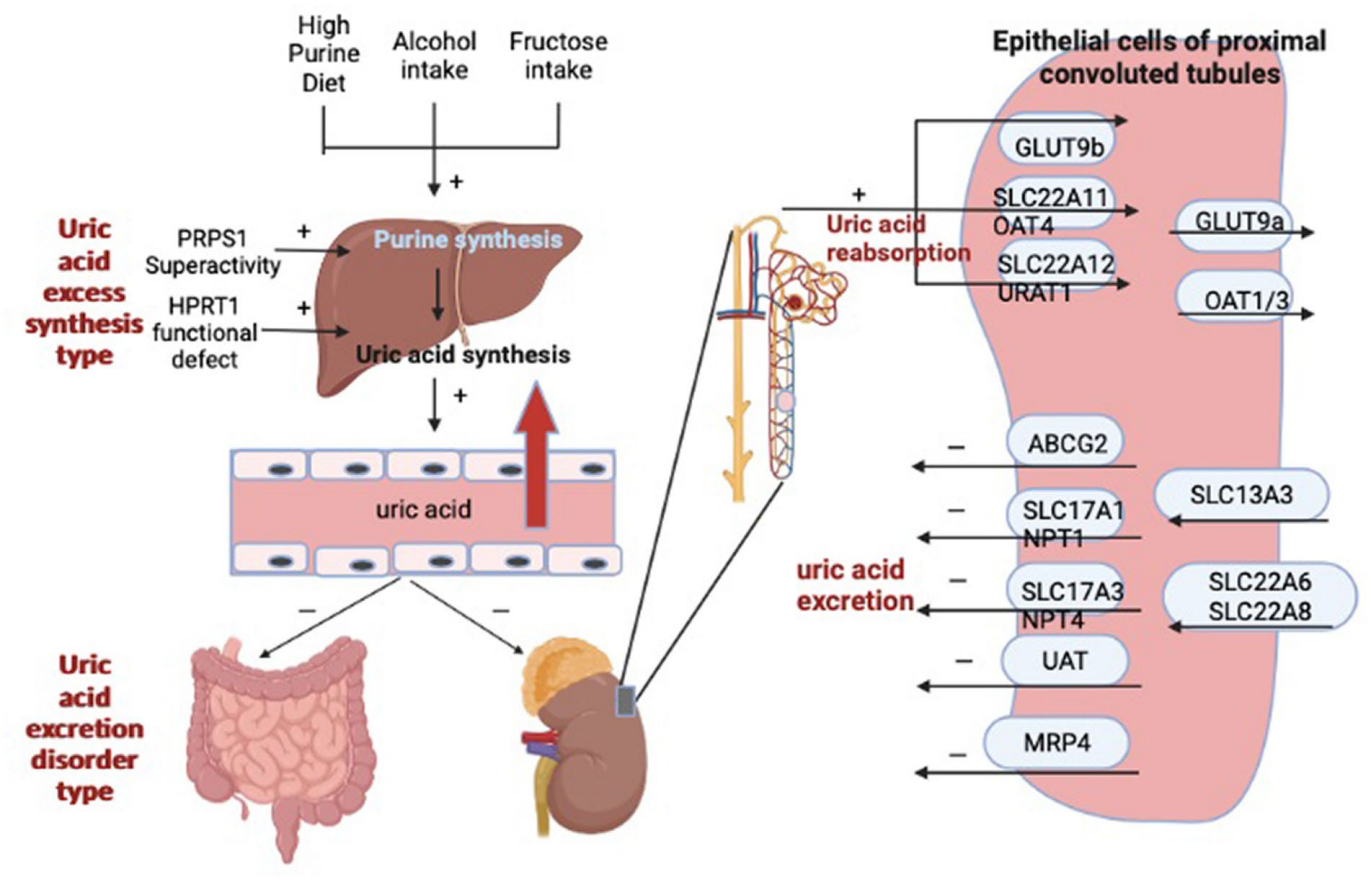
The factors influencing hyperuricemia.

It was found that UA can be oxidized by urate oxidase to 5-hydroxyurate, which in turn is converted to allantoin (8). However, humans and primates have evolved without urate oxidase and are therefore unable to break down uric acid into allantoin. Therefore, most UA is excreted only by the kidneys, with a very small proportion excreted by the intestine, and the excretion of UA often relies on the synergistic action of multiple uric acid transporters. For example, uric acid transporter 1 (URAT1), organic anion transporter 4 (OAT4), and glucose transporter 9 (GLUT9) mainly regulate UA reabsorption, while adenosine triphosphate-binding transporter protein G superfamily member 2 (ABCG2), organic anion transporter 1 (OAT1) and 3 (OAT3) are responsible for regulating renal UA excretion (9–11). It has been demonstrated that xanthine oxidase (XOD) catalyzes the oxidation of hypoxanthine and xanthine to uric acid and is a key enzyme in the control of UA production (12). However, the production and metabolism of UA are complex physiological processes. In addition to XOD, it also involves phosphoribosyl pyrophosphate synthetase (PRPS), hypoxanthine-guanine phosphoribosyl transferase (HGPRT), phosphoribosyl pyrophosphate amino-transferase (PRPPAT) (13). Figure 1 shows the factors affecting hyperuricemia. Therefore, reducing uric acid production by adjusting the activity of uric acid metabolism enzymes, increasing uric acid excretion by promoting the expression of UA transporters, and controlling the intake of foods containing high purine can achieve the goal of reducing uric acid. However, the current treatment for HUA is more through drug therapy, which can be divided into synthesis inhibitors and excretion promoters according to the formation mechanism of UA. The former includes Allopurinol and Febuxostat, the latter includes Propofol and Benzbromarone; oxidative breakdown of uric acid can also be achieved by ingestion of pegloticase and rasburicase. Although the above-mentioned drugs can positively affect the lowering of uric acid, they can also have some harmful effects on the body. It has been found that allopurinol and benzbromarone cause skin sensitization and nephrotoxicity, respectively, in clinical treatment (14, 15).

Therefore, it is necessary and of great importance to find safer and more effective natural drugs to replace HUA therapeutic drugs.

Chinese medicine believes that food and medicine originated at the same time, hence the term medicine and food harmony. Drugs and food are relative: drugs are also food, while food is also drugs; food has fewer side effects, while drugs have more. Medicine and food harmony are scientifically combined for both food and medicine; they meet nutritional needs as well as the function of preventing and treating diseases (16). Interestingly, as research has progressed, many medicinal and edible plants have been linked to a reduction in the incidence of various diseases, such as diabetes, hypertension, and vascular disease (17–19). Therefore, there is an increasingly growing interest in the research and development of medicinal and food plants (20–22). In recent years, functional foods developed from medicinal and edible plants for the treatment of HUA have received greater attention. For example, it is found that chicory extract can significantly reduce serum uric acid levels as well as increase intestinal uric acid excretion in rats with high glucose-induced HUA (*p* < 0.05), which in turn was found to enhance the mRNA expression of ABCG2 and promote uric acid excretion (23). *Nelumbinis folium* leaf extract showed positive inhibition of XOD activity, thus showing potential for alternative drug treatment of HUA (24). This review highlights medicinal and edible plants with UA-lowering activity and their bioactive compounds for potential UA-lowering mechanisms. We expect that this paper will contribute to the understanding of the potential applications of medicinal and edible plants in the prevention and treatment of HUA.

## 2. Medicinal food homologous plant functional food that can reduce uric acid

### 2.1. Chinese quince

Chinese quince (*Chaenomeles sinensis*) is often used in Chinese medicine to treat HUA and gout. Modern pharmacological studies have also shown that it contains many esters, acids, alcohols, aldehydes, ketones, heterocycles, and alkanes (25). Zhang et al. (26) mixed dried Chinese quinces with 30% ethanol and extracted them at 50°C for 1.5 h, followed by centrifugation at 5,000 rmp for 15 min, after which the crude extract was obtained by rotary evaporation of the solvent. Finally, the ethyl acetate fraction of *Chaenomeles sinensis* fruit extract (CSF-E) was obtained by AB-8 macroporous resin. They used the obtained CSF-E to treat HUA mice induced by potassium oxyzinate (PO) and showed that CSF-E significantly reduced XOD activity in the serum and liver of mice (*p* < 0.05). It also reduced serum UA, creatinine (CR), and blood urea nitrogen (BUN) levels, and further analysis revealed downregulation of URAT1 as well as upregulation of OAT1 protein expression in the kidney, which increased UA excretion.

### 2.2. Lemon

Lemon (*Citrus limon*) is one of the best-known and most used species in the citrus genus Citrus, which contains high levels of citric acid, proven to have anti-inflammatory, anti-bacterial, anti-cancer, and anti-parasitic activities (27). A recent study found that 30 mL of freshly squeezed pure lemon juice administered daily for six weeks significantly reduced serum uric acid levels (*p* < 0.01), and the same results were found in PO- induced HUA mice (28). Lin et al. (29) found that the water-soluble extract of lemon significantly reduced the serum uric acid level in PO-induced HUA mice (*p* < 0.05) and hardly damaged the kidney. They further found that it was mainly the potassium citrate contained in lemons that exerted the uric acid-lowering effect. This study suggests that potassium citrate has the potential to be developed as a drug for the treatment of HUA.

### 2.3. Chicory

In Chinese traditional medicine, Chicory (*Cichorium intybus L.*) is considered a diuretic and cholagogue, and its effects are often related to the bioactive substances it contains, such as polyphenols, inulin, oligofructose, and sesquiterpene lactones (30). As previously mentioned, chicory extract enhances uric acid excretion by down-regulating ABCG2 mRNA and up-regulating OAT3 mRNA expression in the kidney (23). Similarly, previous work has found that chicory powder has a positive effect on serum metabolites of UA, lipids, glucose, and abdominal fat deposition in a quail model induced by a purine-rich diet. Jin et al. (31) induced HUA rats by gavage of yeast (15 g/kg/d) and adenine (80 mg/kg/d). They found that chicory extracts enhanced 24 h UA excretion and CR clearance in rats, demonstrating that chicory extract significantly reduced serum UA and CR levels in rats (*p* < 0.05). A decrease in GLUT9 protein expression was also found in rat kidneys and human renal tubular epithelial cells (HKC cells) *in vitro*, demonstrating that chicory extract exerted a blood UA-lowering effect by affecting GLUT9 protein expression. Meng Bian’s team used network pharmacology to predict the possible components and corresponding mechanisms of the anti-HUA effect of chicory (32). They selected quail with the same UA metabolic pathway as humans to establish an HUA model to evaluate the potential anti-HUA mechanism of chicory extract. The results suggest that luteolin and β-sterols in chicory may be the key active components of chicory in lowering UA, and the mechanism involved in the regulation of lipolysis in adipocytes. After treatment with chicory extract, the serum UA levels of quail gradually decreased, and serum total cholesterol (TC), triglyceride (TG), and low-density lipoprotein-cholesterol (LDL-C) levels were significantly reduced (*p* < 0.05).

### 2.4. Tea

Tea (*Camellia sinensis*) originated in China, with coffee and cocoa being known as the world’s three major beverages. With the popularity of tea, more and more research is being done on tea. The various components and functions of tea are also gradually being explored, with the most researched being tea polyphenols and antioxidant properties (33–35). It is the presence of antioxidants in tea that can act as inhibitors of many enzymes, including XOD. It has been found that green tea extract can reduce serum UA levels and modulate UA gene transporters to increase renal uric acid clearance in HUA mice by inhibiting XOD enzyme activity (36). Additional studies have found that ester catechins in tea extracts exert stronger XOD inhibitory activity than non-ester catechins (37, 38). Li et al. (39) jointly verified through *in vivo* and *in vitro* experiments that epigallocatechin gallate (EGCG) showed significant anti-HUA effects by inhibiting XOD activity, reducing serum uric acid levels, enhancing renal OAT1 expression, and decreasing GLUT9 expression in rats. Other studies found that theaflavin was able to reduce serum BUN and CR values by inhibiting adenosine deaminase (ADA) and XOD activities, down-regulating gene and protein expression of GLUT9 and URAT1, and up-regulating gene expression of ABCG2, OCTN1, and OAT2, to improve hyperuricemic symptoms and renal damage in HUA mice (40). Putranty et al. (41) studied the effect of green tea extract on the kidney of HUA rats, and they found that 600 mg/kg of green tea extract reduced malondialdehyde (MDA) and CR levels in the kidney while repairing kidney damage. To investigate the effect of each tea on HUA, Sang et al. (42) used each of these six basic teas (Green Tea, White Tea, Yellow Tea, Oolong Tea, Black Tea, and Dark Tea) in HUA rats and interestingly, the results showed that all except green tea showed a lowering effect of serum UA, while yellow tea significantly improved HUA by modulating inflammatory response, autophagy, and apoptosis (*p* < 0.05). In the introductory section, we mentioned that HUA causes disturbances in the gut microbiota. Wu et al. (43) applied aqueous extracts of six teas (TWE) to HUA mice to study the changes in their UA, and found that a significant decrease in xanthine dehydrogenase (XDH) expression in the liver, enhanced ABCG2, OAT1, and OAT3 expression in the kidney, and reduced URAT1 expression in the kidney occurred after TWE intervention. Enhanced expression of ABCG2 in the gut and improved gut microbiota were also found to occur. There are many studies on tea to lower UA, but we can conclude that the gallic acid, polyphenols and theaflavins contained in tea mainly play the role of inhibiting XOD enzyme activity and lowering UA. The mechanisms involved are simply the down-regulation of the expression of genes involved in uric acid reabsorption, such as GLUT9 and URAT1, and the up-regulation of the expression of genes involved in uric acid transport or excretion, such as ABCG2, OAT1, and OAT3, the inhibition of XOD enzyme activity, and also the anti-HUA effect through the modulation of the inflammatory response or gut microbes (44–46).

### 2.5. Sophora

Flos Sophorae (*Sophora japonica* Linn), the flower of the Chinese scholar tree, has remarkable biological activity and is used in several Asian countries to treat diseases such as bleeding hemorrhoids, hematuria, vomiting of blood, and inflammatory conditions (47). The main bioactive components in Flos Sophorae include kaempferol, quercetin, rutin, and isorhamnetin (48). Wang et al. (49) screened the XOD inhibitors of the main components of the aqueous extract of Flos Sophorae using HPLC-MS/MS and found that isorhamnetin significantly reduced serum uric acid levels and inhibited XOD activity, and also reduced serum CR and BUN levels and protected the kidneys. In addition, molecular docking showed that isorhamnetin binds well to XOD and is a promising XOD inhibitor. In a recent study, Li et al. (50) analyzed the active components in Flos Sophorae and also investigated the type and mechanism of XOD inhibition. They found that the IC50 of quercetin, kaempferol, isorhamnetin, rutin, and gibberellin in Flos Sophorae against XOD were 0.03, 0.11, 0.07, 5.62, 11.48, and 22.13 mg/mL, respectively. Only quercetin, isorhamnetin, and kaempferol were mixed competitive inhibitors and significantly inhibited the fluorescence intensity of XOD (*p* < 0.05).

### 2.6. Lotus leaf

Lotus leaf (*Folium nelumbinis*) is the dried leaf of *Nelumbo nucifera* Gaertn. It is used as a herbal medicine to relieve summer heat and spleen deficiency and diarrhea in traditional Chinese medical knowledge (51). Modern research has also found that lotus leaves can lower blood pressure, blood lipids, and blood sugar, and can be used as a new weight loss strategy (52, 53). Previously, we mentioned that the total alkaloids of lotus leaves could significantly inhibit XOD activity with an IC50 of 3.313 μg/mL. Roemerine were analyzed by UHPLC-Q-TOF-MS and 3D molecular docking to identify them as potential active substances (24). A recent study found that both crude extract and the total alkaloid fraction of lotus leaves showed positive XOD inhibitory effects. They inhibit UA production by reducing the mRNA and protein expression of hepatic XOD, and further analysis revealed that the mechanism is to down-regulate renal GLUT9 and URAT1 expression to inhibit uric acid reabsorption and up-regulate renal OAT1 to promote uric acid excretion (54).

### 2.7. Chrysanthemum

Chrysanthemum flowers (*Dendranthema morifolium*) are not only used as ornamental flowers in urban landscapes but also as a traditional medicine or food for people in some Asian countries (55–57). After a long-term research, more than 190 chemical substances have been isolated and identified from chrysanthemum, mainly flavonoids, terpenoids, and phenolic acids, which exert antioxidant, anti-inflammatory, anti-tumor, as well as antibacterial and uric acid-lowering effects (58–60). In a 2017 Japanese study, researchers found that intake of chrysanthemum extract led to a significant reduction in serum UA after week four in young Japanese men with high serum uric acid (*p* < 0.01) (61). Peng et al. (62) compared the inhibition rates of 11 chrysanthemum extracts on XOD and found that *Chrysanthemum morifolium* Ramat *“Boju”* extract (CBE) showed excellent inhibition. Their analysis revealed that the flavonoid glycosides (luteolin, apigenin, diosgenin, and acacetin, accounting for up to 79.8% of the total) played the main inhibitory role. In addition, administration of CBE to HUA rats revealed a significant decrease in serum UA levels and XOD activity (*p* < 0.05), upregulation of renal ABCG2 expression as well as decreased expression of URAT1 and GLUT9, and reduced serum CR and inflammatory factor levels, demonstrating a protective effect on the kidney. As mentioned earlier, the mechanism of UA lowering may involve adipocyte lipolysis regulation (32). Similarly, in this article by Peng (62), the analysis of serum metabolites using UPLC-ESI-QTQF/MS revealed that CBE prevents the pathological process of HUA by regulating 16 biomarkers related to the metabolism of tryptophan, sphingolipids, glycerophospholipids, and arachidonic acid.

### 2.8. Other medicinal and edible plant resources

Lou et al. (63, 64) found that macroporous resin extract of *Dendrobium officinale* Leaves (DLE) could inhibit HUA by inhibiting XOD and ADA activity in the liver and kidney, reducing uric acid production, regulating URAT1, ABCG2, and GLUT9 expression, and inhibiting NF-κB inflammatory pathway. *Alpinia officinarum* extract and *Pueraria lobata* extract also showed positive inhibition of XOD activity, attenuation of oxidative stress, downregulation of URAT1 and GLUT9 protein expression, and reduction of serum urea and creatinine levels (65, 66). Liang et al. (67) used HPLC-DAD-MS/MS to analyze nine compounds from *Poria cocos*, namely 5-O-caffeoylmangiferine, neo-caffeine, paclitaxel, neo-isocaffeine, isocaffeine, and trans-resveratrol, which were able to inhibit XOD activity. These substances significantly reduced serum tumor necrosis factor (TNF)-α, interleukin-1β (IL-1β), interleukin-6 (IL-6), interleukin-12(IL-12), UA, and BUN (*p* < 0.01), and protected chondrocytes from erosive damage. Chen et al. (68) found a flavonoid extract in saffron (*Crocus sativus*). After treatment of HUA rats with this extract, on the one hand, serum UA, CR, and BUN levels were significantly reduced in HUA rats (*p* < 0.05), serum and liver XOD activities were inhibited, and serum TG and LDL-C levels were significantly reduced (*p* < 0.05). On the other hand, it ameliorated intestinal microbial dysbiosis and disorders of lipid and amino acid metabolites in HUA rats. *Agrocybe aegerita* has a unique taste and function and is used worldwide as food or medicine. Yong et al. (69) extracted *Agrocybe aegerita* with ethanol and water, respectively, and found that they both had an inhibitory effect on hepatic XOD activity and an elevated effect on renal OAT1 expression. 2-Formyl-3,5-dihydroxybenzyl acetate, 2,4-dihydroxy-6-methylbenzaldehyde, 2-(6-hydroxy-1H-indol-3-yl) acetamide and 6-hydroxy-1H-indole-3-carboxaldehyde (HHC) were identified as potential active compounds by molecular docking. It is hypothesized that the potential mechanism is that the active compounds compete with the substrate xanthine for the XOD active site.

From the above summary of 14 common medicinal and food plant resources used in traditional medicine for the treatment of HUA in some Asian countries, it is easy to find that some plant extracts are, they can be used to treat HUA and prevent kidney inflammation by inhibiting XOD activity and lowering serum UA, CR, and BUN levels. At a deeper level, by down-regulating the expression of proteins that promote uric acid reabsorption, such as GLUT9 and URAT1, and up-regulating the expression of proteins that promote uric acid excretion, such as ABCG2, OAT1, OAT2, and OAT3, the anti-HUA is mechanistically achieved by regulating signaling pathways such as lipid metabolism and inflammatory response. And most of these extracts are flavonoids and phenolic substances, such as luteolin, catechins, and isorhamnetin, which play a major role in lowering uric acid and inhibiting XOD activity. Therefore, we next further summarized these bioactive substances with uric acid-lowering effects and their uric acid-lowering mechanisms.

## 3. Bioactive substances and their uric acid-lowering mechanism

In 1806, morphine with analgesic effects was successfully isolated from plants and is also considered the first active ingredient isolated from plants in the world (70). Over the next few hundred years, scientists have continuously isolated active ingredients from plants and studied their pharmacological effects and mechanisms, leading to the continuous development of modern drug innovation. It has been confirmed that plant-active ingredients have a wide range of biological activities. Table 1 shows some biologically active ingredients and their effects. In our previous summary of the various types of medicinal and edible plants, we briefly mentioned that some of these active substances can exert anti-HUA effects by inhibiting XOD activity and lowering serum uric acid levels (87). Therefore, in the next section, we will focus on summarizing the anti-hyperuricemia mechanism of various bioactive ingredients.

**Table 1:** Effects of active ingredients from different sources and its identification methods.

Active ingredients		Sources	Identification methods	Effects	References
Flavonoids	Kaempferol, luteolin, myricetin and (+)-taxifolin	*Mandragora autumnalis* Fruit	^1^HNMR and ^13^C NMR	Anti-diabetic, Anti-lipase, Free radical scavenging activity, Antimicrobial properties	(71)
	Quercetin	*Ziziphus spina-christi* L Fruit	HPLC and GC–MS	Antimicrobial properties	(72)
	Astilbin, morin and naringenin	Leaves of *Lithocarpus polystachyus* Rehd	Affinity separation-UPLC-Q-TOF-MS/MS	Inhibitory activity against α-glucosidase	(73)
	N-nornuciferine, Nuciferine, 2-Hydroxy-1-methoxyaporphine and Isorhamnetin 3-O-glucoside	*Nelumbo nucifera* leaves	Bioaffinity ultrafiltration with multiple targets coupled with HPLC–MS/MS	Exhibited inhibitory activities against α-glucosidase, pancreatic lipase and Cyclooxygenase-2	(53)
Phenolic acids	Gallic acid and ellagic acid	*Ziziphus spina-christi* L Leaves	HPLC and GC–MS	Antimicrobial properties	(72)
	2-methoxy-4-vinylphenol	*Nasturtium officinale* R. Br	UV–Vis, FTIR spectrophotometry and GC–MS	Antioxidant	(74)
	Chlorogenic acid	*Smilax china* L.	HPLC and HPLC–MS	Inhibit XOD activity	(75)
	Chlorogenic acid	Apple pomace	UPLC–Q–Exactive Orbitrap/MS	Antioxidant	(76)
	Chlorogenic acid and p-coumaric acid	Spent coffee grounds	cLC-DAD and LC-MS/MS	Antioxidant	(77)
Alkaloids	Vindoline, vindorosine, vincristine and norseredamine	*Catharanthus roseus* leaves	HPLC-DAD, UPLC-ESI-QTOF-MS/MS and ^1^H-NMR	Anti-cancer	(78)
	Cardioquin	The roots of *Stemona mairei* (H.Lév.)K.Krause	UPLC-IT-TOF, ^1^HNMR and ^13^C NMR	exhibited nematocidal activity	(79)
	Actinidine and glaziovine	*Nardostachys jatamansi*	LC–MS/MS	Anti-neurodegenerative diseases (NDDs)	(80)
Polysaccharide	water-soluble polysaccharide in *G. lucidum* spore (GLSP-I)	*Ganoderma lucidum* spore (*G.lucidum*)	GC–MS	Immunomodulatory activity	(81)
	*Lactarius vellereus* Fr. polysaccharide (LV-1) and *Cordyceps militaris* (L. ex Fr.) Link. polysaccharide (CM-S)	*Lactarius vellereus* Fr. and *Cordyceps militaris* Link	GC–MS, HPGPC, HPLC, FTIR and NMR	Immunoactivity	(82)
	*Hovenia dulcis* polysaccharides (HDPs)	*Hovenia dulcis* (Guai Zao)	FTIR, periodate oxidation, Smith degradation, methylation analysis, and NMR	Hypoglycemic activity	(83)
Saponin	Ginsenosides	*Panax ginseng* Meyer (Korea red ginseng (KRG))	HPLC	Anti-inflammatory	(84)
	Dammarane-type triterpenoid saponins	The roots of Panax notoginseng	HPLC	Anti-inflammatory, anti-angiogenetic and anti-dengue virus	(85)
	Ternstroenol F	*Ternstroemia cherryi*	^1^H NMR	Anti-inflammatory	(86)

NMR, Nuclear Magnetic Resonance; HPLC, High Performance Liquid Chromatography; GC–MS, Gas Chromatography-Mass Spectrometer; UPLC-Q-TOF-MS/MS, Ultra High Performance Liquid Chromatography Four Stage Rod Time of Flight Tandem Mass Spectrometry; HPLC-MS/MS, High performance liquid chromatography-mass spectrometry; UV–Vis, Ultraviolet visible; FTIR, Fourier Transform Infrared Spectroscopy; cLC-DAD, High performance liquid chromatography system; LC–MS/MS, Liquid Chromatograph Mass Spectrometer; HPLC-DAD, High-performance liquid chromatography-diode array detection; UPLC-ESI-QTOF-MS/MS, Ultra performance liquid chromatography electrospray ion source quadrupole time-of-flight mass spectrometry; UPLC-IT-TOF, Ultra Performance Liquid Chromatography ion trap time-of-flight; HPGPC, High Performance Gel Permeation Chromatography.

### 3.1. Flavonoids

Flavonoids generally refer to a series of compounds formed by the interconnection of two benzene rings with phenolic hydroxyl groups through central three carbon atoms. It is among the most studied natural compounds and has received a lot of attention for its uric acid-lowering effects (88, 89). In a previous study, researchers selected 15 flavonoids to study their uric acid-lowering effects in HUA mice. It was found that continuous oral administration of 50 and 100 mg/kg of quercetin, mulberry pigment, kaempferol, apigenin, and gerberinol for 3 days significantly reduced hepatic uric acid levels and significantly inhibited hepatic xanthine oxidase activity in mice (90) (*p* < 0.01).

Yuan et al. (91) extracted and identified flavonoid components from corn beard and found that the flavonoid components eluted from 60% ethanol solution had the best uric acid-lowering effect. It could reduce serum uric acid level by 26.69% and XOD activity by 11.29%. It was also hypothesized that apigenin-6-C-glucoside-7-O-glucoside, kaempferol-3-globoside, lignan-7-glucoside, lignan-3′,7-di-O-glucoside and naringenin among them would affect the XOD activity.

Quercetin is one of the most abundant flavonoids in the daily diet and has been studied as an anti-cardiovascular, anti-cancer, anti-viral, and antioxidant drug (92–96). In recent years, studies have found that quercetin has the potential to treat HUA, and as a result, more and more research has been conducted on quercetin for the treatment of HUA (97). Zhang et al. (98) investigated the dual-substrate kinetics and inhibition mechanism of quercetin on XOD by combining kinetic analysis, molecular docking techniques, and multispectral approaches. The IC50 of quercetin against XOD was found to be 2.74 ± 0.04 mol/L. It also reversibly inhibited the production of uric acid and O2- in a mixed manner, and the binding process with XOD was mainly dominated by van der Waals forces and hydrogen bonds. In a study at the University of Leeds, Sarka et al. (99) found that quercetin and its metabolites were able to inhibit the activity of xanthine oxidoreductase (XOR, one of the key rate-limiting enzymes of purine metabolism) exerting a lowering effect on uric acid *in vivo*. Also in a study by Yu et al. (45), quercetin was found to show relatively strong URAT1 inhibitory activity with an IC50 of 12.6 μM. Zhu et al. (100) found that quercetin derivatives (quercetin-3-O-β-D-galactopyranoside, quercetin-3-O-γ-D-glucopyranoside, and quercetin-3-O-α-L-rhamnoside) inhibited XOD activity and reduced the expression of intracellular reactive oxygen species (ROS), IL-1β, IL-1β, and NOD-like receptor superfamily containing 3 (NLRP3) inflammatory vesicles, showing significant therapeutic effects on HUA and renal inflammation.

Isorhamnetin is a dimethylated flavonol that is found in sea buckthorn and ginkgo leaves (101). In a previous study by Wang (49), isorhamnetin has been shown to significantly reduce serum uric acid levels and inhibit hepatic XOD activity in mice. Also in a study by Adachi et al. (102), they found that oral administration of isorhamnetin reduced uric acid levels in blood and liver in a dose-dependent manner and decreased XOD activity. Luteolin is a natural flavonoid found in a variety of plants and has a wide range of pharmacological effects, such as anti-inflammatory, anti-tumor, anti-viral, and uric acid-lowering (103–105). Back in 2013, the team of Yan (106) found that luteolin reversibly competitively inhibited XOD with an inhibition constant (Ki) value of (2.38 ± 0.05) × 10–6 mol/L. They found that luteolin interacts with amino acid residues of the first order within the active site pocket of XOD by fluorescence and circular dichroism. In a 2017 clinical trial in Japan, researchers found that a continuous intake of 10 mg of luteolin for four weeks was able to reduce serum uric acid levels (61). Lin et al. (107) also investigated the therapeutic effects of luteolin and luteolin-4’-O-glucoside in HUA mice. The results revealed that both showed positive anti-HUA effects by decreasing URAT1 levels and inhibiting XOD activity. The levels of IL-1β and TNF-α were also reduced to improve inflammation. Rajesh et al. (108) extracted luteolin from millet and found a binding energy of −9.7 kcal/mol with XOR by molecular docking, while the binding energy of allopurinol with XOR was −8.0 kcal/mol, indicating the potential of luteolin as an XOR inhibitor. It has been found that luteolin inhibited XOD activity and mRNA expression of XDH while inducing increased expression levels of OAT1 and OAT3 proteins as well as decreased GLUT9 and URAT1 expression. In addition, in a study by Zhu et al. (109), it was found that luteolin reversed the decrease in OAT1 levels and inhibited XOD activity as well as TLR4/MyD88/NLRP3 signaling pathway in the liver.

Overall, flavonoids reduce uric acid production by inhibiting XOD and XOR enzyme activities and regulating the expression of GLUT9, UART1, OAT1, and other related proteins. It also promotes uric acid excretion to reduce HUA and reduces the expression of inflammatory vesicles such as IL-1β, IL-1β and NLRP3 inflammatory vesicles and TLR4/MyD88/NLRP3 signaling pathway to protect the kidney or joints from inflammatory damage. The established hypouricemic mechanisms of flavonoids in bioactive substances are summarized in Table 2.

**Table 2:** Anti-hyperuricemic effect of flavonoids from different sources.

Source	Active ingredient	Model	Dose	Mechanism	References
*Ginkgo biloba* leaves	Quercetin, apigenin, kaempferol and isorhamnetin	XOD model *in vitro*	\	↓ XOD activity	(110)
*Sophora japonica*	Isorhamnetin	PO and hypoxanthine induced HUA mice	50, 100, and 150 mg/kg	↓UA, XOD activity, CR, BUN	(49)
*Cyclocarya paliurus*	Quercetin-3-O-β-D-galactopyranoside, quercetin-3-O-β-D-glucopyranoside, and quercetin-3-O-α-L-rhamnopyranoside	PO induced HUA mice	0.378 and 1.140 g/kg	↓UA, XOD activity, NLRP3 and IL-1β	(100)
*Aspalathus linearis*	Quercetin	Human embryonic kidney 293-derived 293A cells	\	↓ URAT1	(45)
*Sophora japonica* flower bud	Hydroxygenkwanin, Genistein, Tectorigenin, Kaempferol, Sophoricoside and Quercetin	PO and hypoxanthine induced HUA mice	100 mg/kg	↓UA, XOD activity and BUN	(111)
*Sophora japonica*	Quercetin, kaempferol, and isorhamnetin	XOD and ADA model *in vitro*	0.025 mg/mL to 1.0 mg/mL	↓ XOD and ADA activity	(112)
Corn silk	Apigenin-6-C-glucoside-7-O-glucoside, kaempferol-3-O-rutinoside, luteolin-7-glucoside, luteolin-3′,7-di-O-glucoside, and naringenin	PO induced HUA mice	20 mg/kg	↓ UA and XOD activity	(91)
*Fagopyrum tataricum*	Quercetin and kaempferol	XOD model *in vitro*	\	↓ XOD activity	(113)

### 3.2. Phenolic acids

Phenolic acids are a class of organic acids containing phenolic rings and are one of the main types of polyphenols (114). Phenolic acid compounds refer to a class of compounds with several hydroxyl groups on the same benzene ring, and there are two main types of corresponding carbon frameworks. One type is C6-C1 type, which is benzoic acid type, such as p-hydroxybenzoic acid, gallic acid, protocatechu, and the other type is C6-C3 type, which is cinnamic acid type, such as caffeic acid, ferulic acid, p-coumaric acid, and mustard acid (115). It has been shown that Phenolic acids have numerous health benefits suc*h as* antioxidant properties, anti-inflammatory, antibacterial, and neuroprotective effects (115–117). In recent years, phenolic acid compounds have been found to have XOD inhibitory activity and have been suggested to have potential effects against HUA. Lin et al. (118) identified 10 phenolic acid compounds from *Coix lacryma* bran-free polyphenol extract by UPLC-QTOF-MS/MS. For the first time, mustard acid was found to inhibit XOD in a mixed non-competitive manner. In a recent study, researchers compared free polyphenols in *Lactobacillus acidophilus* NCUF202.2 fermented *artemisia selengensis* turcz extracts (FASTE) and *artemisia selengensis* turcz (AST) and the therapeutic effects on HUA rats. The results revealed that coffee quinic acid in FASTE significantly increased the content of free polyphenols (*p* < 0.05), enhanced the inhibitory effect on XOD, and better reduced serum ADA activity. And it increases the expression of uric acid secretory protein OAT1 and decreases the expression of reabsorption proteins URAT1 and GLUT9 in model rats, thus reducing serum uric acid levels in model rats (119).

Chlorogenic acid is an ester produced by caffeic acid and quinic acid (1-hydroxyhexahydro gallic acid), a phenylpropanoid compound produced by the plant body during aerobic respiration via the mangiferous acid pathway (120, 121). Zilma et al. (122) found that the aqueous extract of Tabebuia roseoalba leaves had an inhibitory effect on hepatic XOD and reduced serum uric acid levels. Further identification by HPLC analysis revealed that caffeic and chlorogenic acids may be responsible for the activity exhibited by the extract. In a study by Zhou et al. (123), chlorogenic acid was found to decrease UA, BUN, CR levels and inhibited XOD activity in hypoxanthine and oxokalate induced HUA mice. Chlorogenic acid also down-regulated the mRNA expression of UA secreted proteins. In addition, chlorogenic acid significantly reduced the mRNA expression of IL-1β, TNF-α, NLRP3 and Caspase-1 (*p* < 0.05), inhibited TLR4/MyD88/NF-κB signaling pathway in the kidney and attenuated renal inflammation in HUA mice.

Caffeic acid is also one of the products of the phytocannabinoid pathway. Extensive studies have confirmed the biological activities of caffeic acid such as anti-microbial, anti-cancer, antioxidant and anti-inflammatory (124–127). In a study by Wan et al. (128), PO-induced gavage of caffeic acid (100 mg/kg) for 7 days in HUA rats significantly reduced serum uric acid and XOD activity (*p* < 0.01). Caffeic acid achieves its uric acid-lowering effect mainly by down-regulating the transcript levels of URAT1 and GLUT9 mRNAs and up-regulating the transcription of OAT1, UAT and ABCG2 mRNAs. *In vitro* studies showed that caffeic acid was competitively inhibited with XOD, with an IC50 value of 53.45 μM. Foreign researchers studied ethanolic extract of Lobelia and found that its ethanolic extract and its components (chlorogenic and caffeic acids, etc.) had inhibitory activity on hepatic XOD and were able to lessen the size of paw edema caused by urate crystals (129).

Gallic acid is a polyphenolic compound widely found in fruits, plants and nuts, with a variety of biological activities including anti-inflammatory, anti-mutagenic, antioxidant and anti-cancer (130–132). Wu et al. (37) established a HUA model using normal human hepatocytes (HL-7702). They found that gallic acid significantly inhibited uric acid production at the cellular level (*p* < 0.05), and the inhibition was stronger than that of tea polyphenols and theaflavins. Chinese researchers identified the active compound gallic acid (GA) by HPLC from ethyl acetate extracts of *Sonneratia apetala* leaves and branches and investigated its mechanism of regulating uric acid metabolism in HUA mice (38). It was found that GA inhibited ADA and XOD activities and was able to down-regulate the expression of URAT1 and GLUT9 proteins, up-regulate the expression of ABCG2 protein as well as inhibit MDA, IL-6, IL-1β, TNF-α and enhance the activity of SOD and GSH-Px in the kidney. These suggest that GA reduces the levels of uric acid and prevents renal injury by inhibiting oxidative stress and inflammation and regulating the expression of uric acid transporters in HUA mice.

Phenolic acids had inhibitory activity against ADA and XOD, increased the expression of uric acid secretory proteins (OAT1, UAT, and ABCG2) and inhibited uric acid reabsorption proteins (URAT1 and GLUT9). It also inhibited the activity of oxidative stress and related inflammatory bodies (IL-1β, TNF-α, NLRP3, and Caspase-1), and exerted the effect of lowering uric acid level to prevent HUA and protect the kidney from inflammatory damage. The established hypouricemic mechanisms of phenolic acids in bioactive substances are summarized in Table 3.

**Table 3:** Anti-hyperuricemic effect of phenolic acids from different sources.

Source	Active ingredient	Model	Dose	Mechanism	References
Coix seed	Dihydroferulic acid	PO and hypoxanthine induced HUA mice; XOD model *in vitro*	500 mg/kg	↓ UA, CR and XOD activity	(133)
Purple potato leaves	Caffeoylquinic acid	PO and hypoxanthine induced HUA mice		↓ UA, CR, XOD and ADA activity ↓ IL-1β, IL-6, and TNF-α	(134)
*Chrysanthemum morifolium*	Caffeoylquinic acid	PO induced HUA mice	25 and 50 mg/kg	↓ UA and XOD activity; ↓ Xanthine and hypoxanthine levels	(135)
*Sonneratia apetala* leaves and branches	Gallic acid	PO and hypoxanthine induced HUA mice	25, 50 and 100 mg/kg	↓ UA, CR, BUN; XOD and ADA activity; MDA, IL-6, IL-1β, TNF-α, Cyclooxygenase-2 (COX-2) and cystatin-C (Cys-C); ABCG2; ↑ URAT1 and GLUT9; SOD, GSH-Px and Na^+^–K^+^–ATPase	(38)

### 3.3. Alkaloids

Alkaloids are a category of nitrogen-containing alkaline organic compounds found in nature with significant biological activities, such as anti-cancer and anti-bacterial (136–138). It was found that the total lotus leaves alkaloids showed positive inhibition of XOD enzyme activity with an IC50 value of 3.313 μg/mL, and UHPLC-Q-TOF-MS and 3D docking analysis revealed lotus alkaloids as potential active ingredients (24). Not only that, in PO-induced HUA mice, lotusine reduced serum uric acid levels by a possible mechanism of reversing the expression of related uric acid transporters (URAT1, GLUT9, ABCG2) as well as inhibiting TLR4/MyD88/NF-κB signaling pathway and NLRP3 inflammatory vesicles activation in the kidney, exerting anti-HUA and anti-inflammatory effects (139).

Berberine (BBR), also known as flavopiridol, is a quaternary ammonium alkaloid isolated from the Chinese medicine Huanglian, which has a wide range of antibacterial activity (140). It was found that berberine significantly reduced uric acid levels in the blood and feces of HUA rats (*p* < 0.05), while blood XOD levels decreased, ABCG2 levels increased and Galectin-9 levels decreased (141). Li et al. (142) found 82 targets of berberine acting on HUA as well as several inflammation-related signaling pathways by network pharmacology prediction. *In vivo* experiments revealed that berberine significantly reduced UA, BUN and CRE levels in HUA mice (*p* < 0.05), decreased IL-1β and IL-18 levels, and down-regulated the expression of URAT1, NLRP3, apoptosis-associated spot-like protein (ASC), Caspase1 and IL-1β, showing anti-HUA and prevention of renal effects on kidney injury. Berberine also reduced hepatic XOD activity at the protein and mRNA levels, down-regulated GLUT9 expression, up-regulated OAT1/3 and ABCG2 expression, and inhibited the JAK2/STAT3 signaling pathway to exert anti-HUA effects (143, 144). Zhong et al. (145) used PO and hypoxanthine to induce HUA mouse models for 7 consecutive days, followed by gavage of oxyberberrubine (oxyberberrubine is a metabolite of berberine in the liver, 5, 10 and 20 mg/kg), while febuxostat (Fex, 5 mg/kg) was used as a positive control. After OBR treated, the levels of serum uric acid was reduced and inhibition of XOD and ADA activities were enhanced, along with decreased expression of XOD, URAT1, GLUT9, NLRP3, ASC and Caspase-1 at the mRNA and protein levels; it also significantly reduced serum creatinine and blood urea nitrogen and inflammatory mediators (TNF-α, IL-1β, IL-6 and IL-18) levels (*p* < 0.05).

Alkaloids reduce XOD and ADA activity by inhibiting XOD expression in the liver. It also downregulates the expression of GLUT9 and URAT1, upregulates the expression of ABCG2 and OAT1/3, reduces serum uric acid levels, prevents HUA as well as inhibits the expression of inflammatory vesicles and inflammatory signaling pathways, and protects the kidney from inflammatory damage. The established hypouricemic mechanisms of alkaloids in bioactive substances are summarized in Table 4.

**Table 4:** Anti-hyperuricemic effect of alkaloids from different sources.

Source	Active ingredient	Model	Dose	Mechanism	References
*Tuber melanosporum*	Tuberindine A	The monosodium urate (MSU) -induced HK-2 cell model	\	↓ UA levels ↑ OAT1 and ABCG2	(146)
*Folium nelumbinis*	Nuciferine	PO induced HUA mcie	25 and 250 mg/kg	↓ UA levels	(147)
The leaves of *Nelumbo nucifera*	Nuciferine	PO induced HUA mice	10, 20 and 40 mg/kg	↓ UA, URAT1, GLUT9; Toll-like receptor 4/myeloid differentiation factor 88/NF-kappaB (TLR4/MyD88/NF-κB) signaling and NOD-like receptor family, pyrin domain containing 3 (NLRP3); IL-1β; ↑ ABCG2,	(139)

### 3.4. Polysaccharides

Polysaccharide is formed by condensation and water loss of several monosaccharide molecules and is a class of sugar substances with complex and large molecular structure, which has various biological activities such as anti-cancer, anti-bacterial, anti-inflammatory and anti-HUA (148–151). It can be divided into homopolysaccharides and heteropolysaccharides. The former includes starch, glycogen, and cellulose, while the latter includes hyaluronic acid, etc. *Lonicera japonica* polysaccharide (LJP-1) has anti-uricemia and anti-gout effects on PO and hypoxanthine induced HUA rats. The mechanism alleviated the degree of ankle joint swelling in a sodium urate crystal-induced gouty arthritis model by inhibiting XOD activity and reducing serum uric acid levels, IL-1β, IL-6, TNF-α, and COX-2-related inflammatory factor levels (152). Yu et al. (153) established a HUA model by intraperitoneal injection of PO (250 mg/kg) into eight-week-old C57BL/6 male mice for seven consecutive days and treated with a dose of *Lycium barbarum* polysaccharide (LBP) for seven consecutive days. The results revealed that 100 and 200 mg/kg of LBP significantly reduced serum uric acid levels and elevated urinary uric acid levels (*p* < 0.05). It also significantly reduced XOD activity in serum and liver (*p* < 0.01). It also increased the mRNA and protein expression levels of OAT1/3 as well as decreased GLUT9 levels. Ganoderma lucidum polysaccharide (GP) significantly reduced UA, BUN and alaninetransaminase (ALT) levels by inhibiting hepatic and blood ADA activity (*p* < 0.05) and increased UA excretion by inhibiting GLUT9 and OAT1 expression (154, 155).

In general, polysaccharides are macromolecules with low toxicity (156, 157), which can be widely used in the development of various functional foods. Polysaccharides also have a therapeutic effect on HUA by a mechanism related to the inhibition of XOD and ADA activity to reduce UA production. It is also associated with the enhancement of OAT1/3 and inhibition of GLUT9 protein expression to promote UA excretion as well as protection of the kidney and joints from inflammatory damage by reducing the levels of IL-1β, IL-6, TNF-α and COX-2-related inflammatory factors.

### 3.5. Saponins

Saponins are composed of saponins and sugars, which can be divided into steroidal saponins and Triterpenoid saponin. It is mainly found in terrestrial higher plants and small amounts in marine organisms such as starfish and sea cucumbers (158, 159). Studies have shown that saponins have biological activities such as antioxidant and anti-inflammatory (160, 161).

The total saponin content of natural plant “*Yaocha”* extract is 1.0 to 1.5%, and the saponin content of *Camellia sinensis* is 0.4 to 0.8%. Its inhibition of XOD activity by upregulating the mRNA expression of hypoxanthine guanine phosphoribosyltransferase transferase (HPRT1) and OAT1 makes “*Yaocha”* a potential therapeutic agent for the treatment of HUA (162). The total saponin of Huangshan yam was extracted using 70% ethanol at 80°C, and its effects on serum uric acid level (SUA), urinary uric acid level (UUA), uric acid clearance (CUR) and creatinine clearance (CCR) as well as the expression of related uric acid transporters were investigated in HUA rats within 24h (163). The results revealed that saponin administration was dose-dependent, with increased UUA, CUR and CCR values and decreased SUA within 24 h; and decreased mRNA and protein expression of URAT1 and GLUT9 and increased OAT1/3 expression in the kidney.

In general, saponins have similar mechanisms of HUA inhibition as most plant active ingredients. Saponins reduce UA levels and increase UA excretion by inhibiting the activity of XOD, a key enzyme for uric acid synthesis. It also reduced the expression of URAT1 and GLUT9 in the kidney, promoted the expression of ABCG2 to reduce serum uric acid levels, and downregulated NLRP3 inflammatory vesicles to have a therapeutic effect on gouty arthritis (164, 165). The established hypouricemic mechanisms of saponins in bioactive substances are summarized in Table 5.

**Table 5:** Anti-hyperuricemic effect of saponins from different sources.

Source	Active ingredient	Model	Dose	Mechanism	References
*Panax ginseng* C. A. Meyer	Ginsenosides	PO and adenine induced HUA mice	20 mg/kg	↓ UA, CR levels	(166)
*Smilax riparia*	Pallidifloside D	PO induced HUA mice	5, 10 and 20 mg/kg	↓ UA, URAT1 and GLUT9	(164)

## 4. Conclusion

HUA has become a global public health hazard after chronic diseases such as “three highs” and diabetes, and its incidence is increasing rapidly worldwide. Traditionally, drugs for the treatment of HUA have been shown to cause a series of side effects on the human body, thus limiting the clinical treatment of HUA. Therefore, medicinal plants have been extensively studied by researchers working in medicine worldwide for their significant uric acid-lowering effects and are considered as a future therapeutic alternative to HUA. In this regard, researchers are intrigued by the large number of bioactive components contained in medicinal plants. On this basis, numerous experimental studies were conducted in various countries to understand the uric acid-lowering mechanisms of these active ingredients, leading to the discovery that active ingredients such as flavonoids, phenolic acids, and alkaloids inhibit uric acid production or promote uric acid excretion. Up to now, although a huge number of *in vivo* and *in vitro* experiments have shown that medicinal plants have significant therapeutic effects on HUA, the current research is still limited. We know that not only XOD plays a role in the production of uric acid, but also PRPS, HGPRT, and PRPPAT play indispensable roles in uric acid metabolism. However, there is currently limited research on the mechanism of action of plant extracts on uric acid metabolism enzymes, with most studies focusing on the related enzymes (ADA and/or XDH) and the expression of uric acid transporters during the process of uric acid production. Meanwhile, many of these medicinal plants have been extracted to obtain their crude extracts or to isolate certain active ingredients, but these studies have been limited to the laboratory stage only and have never been studied clinically, nor have toxicological studies been performed. In addition, the metabolic pathway of uric acid in humans is significantly different from that in rodents. The possible differences are undeniable as the enzyme uricase, which converts uric acid into allantoic acid, does not exist in humans, whereas rodents can convert uric acid into allantoic acid and excrete it through uricase.

Although there are still some pressing problems in the treatment of HUA with medicinal plants, it does not prevent it from being a potential drug for the prevention and treatment of HUA in the future. In previous studies, we found that molecular docking techniques could be used to elucidate the relationship between bioactive components and XOD and to determine the mechanism of inhibition in this way. In subsequent studies, we can use network pharmacology and molecular docking techniques to explore the binding between bioactive ingredients and HUA targets and uric acid transporters and uric acid metabolizing enzyme and use them as evidence of the effectiveness of bioactive ingredients against HUA. In conclusion, medicinal plants are available as an effective strategy for the prevention and treatment of HUA, and the active ingredients in them provide new directions for the synthesis of effective and less toxic drugs for the treatment of HUA, indicating the potential of medicinal plants for the prevention and treatment of HUA in the future.

## Author contributions

DJ-g is responsible for writing and revising the thesis. WC-y is responsible for the revision and guidance of the thesis. All authors contributed to the article and approved the submitted version.

## Funding

This study was supported by Chongqing Science and Technology Project, cstc2016zdcy-ztzx10001.

## Conflict of interest

The authors declare that the research was conducted in the absence of any commercial or financial relationships that could be construed as a potential conflict of interest.

## Publisher’s note

All claims expressed in this article are solely those of the authors and do not necessarily represent those of their affiliated organizations, or those of the publisher, the editors and the reviewers. Any product that may be evaluated in this article, or claim that may be made by its manufacturer, is not guaranteed or endorsed by the publisher.
